# Interactions between tumor-associated macrophages and regulated cell death: therapeutic implications in immuno-oncology

**DOI:** 10.3389/fonc.2024.1449696

**Published:** 2024-11-07

**Authors:** Yifei Ge, Lixue Jiang, Chengru Yang, Qingfu Dong, Chengwu Tang, Yi Xu, Xiangyu Zhong

**Affiliations:** ^1^ Department of Hepatopancreatobiliary Surgery, The Second Affiliated Hospital of Harbin Medical University, Harbin, Heilongjiang, China; ^2^ Department of Breast Surgery, The Second Affiliated Hospital of Harbin Medical University, Harbin, Heilongjiang, China; ^3^ Department of Hepatopancreatobiliary Surgery, Huzhou Key Laboratory of Translational Medicine, First Affiliated Hospital of Huzhou University, Huzhou, Zhejiang, China; ^4^ Key Laboratory of Clinical Laboratory Diagnosis and Translational Research of Zhejiang Province, The First Affiliated Hospital of Wenzhou Medical University, Wenzhou, Zhejiang, China; ^5^ State Key Laboratory of Targeting Oncology, National Center for International Research of Bio-targeting Theranostics, Guangxi Key Laboratory of Bio-targeting Theranostics, Collaborative Innovation Center for Targeting Tumor Diagnosis and Therapy, Guangxi Medical University, Nanning, China; ^6^ Fujian Provincial Key Laboratory of Tumor Biotherapy, Fuzhou, Fujian, China; ^7^ Fujian Provincial Key Laboratory of Translational Cancer Medicine, Fuzhou, Fujian, China; ^8^ Department of Pathology, Li Ka Shing Faculty of Medicine, The University of Hong Kong, Hong Kong, Hong Kong SAR, China

**Keywords:** tumor-associated macrophages, regulated cell death, tumor microenvironment, ferroptosis, pyroptosis, necroptosis, apoptosis, cancer therapy

## Abstract

Tumor-associated macrophages (TAMs) play a pivotal role in sculpting the tumor microenvironment and influencing cancer progression, particularly through their interactions with various forms of regulated cell death (RCD), including apoptosis, pyroptosis, ferroptosis, and necroptosis. This review examines the interplay between TAMs and these RCD pathways, exploring the mechanisms through which they interact to promote tumor growth and advancement. We examine the underlying mechanisms of these intricate interactions, emphasizing their importance in cancer progression and treatment. Moreover, we present potential therapeutic strategies for targeting TAMs and manipulating RCD to enhance anti-tumor responses. These strategies encompass reprogramming TAMs, inhibiting their recruitment, and selectively eliminating them to enhance anti-tumor functions, alongside modulating RCD pathways to amplify immune responses. These insights offer a novel perspective on tumor biology and provide a foundation for the development of more efficacious cancer therapies.

## 1 Introduction

The tumor microenvironment (TME) is a highly dynamic and intricate ecosystem that surrounds tumor cells. It comprises a wide array of cellular and non-cellular elements, including immune cells, fibroblasts, endothelial cells, extracellular matrix (ECM), cytokines, and growth factors. These components interact extensively, collectively shaping tumor behavior, progression, and response to treatment (1, 2). Tumor-associated macrophages (TAMs), derived from circulating monocytes, constitute a significant proportion of the tumor’s immune cell population and are critical components of the TME. Their notable plasticity and dual role in tumor biology—either promoting or inhibiting tumor growth depending on their polarization state—make them a central focus of cancer research and therapeutic strategies (3, 4). In numerous malignancies, including lung, breast, hepatocellular carcinoma, gastric, and melanoma, TAMs are frequently associated with disease progression, metastasis, and poor outcomes (5–10). Notably, TAMs derived from tissue-resident macrophages engage in efferocytosis, the process of clearing apoptotic cancer cells, particularly during aggressive tumor growth (11). This clearance typically occurs within an immunosuppressive TME, leading TAMs to adopt an anti-inflammatory profile, which ultimately contributes to a tumor-promoting effect (12). However, during immunogenic chemotherapy, radiotherapy, or active immunotherapy, tumor cell death can reprogram TAMs to exert anti-tumor effects (13).

Regulated cell death (RCD) encompasses a range of genetically programmed processes that lead to cell death, playing a fundamental role in maintaining tissue homeostasis and eliminating damaged or unwanted cells (14). The major types of RCD include apoptosis, necroptosis, pyroptosis, and ferroptosis, each characterized by distinct biochemical and molecular pathways (15, 16). RCD pathways are crucial in predicting patient survival, cancer progression, metastasis, and in monitoring the immune system’s engagement in cancer (17–19). Increasing evidence suggests that different forms of RCD can alter the TME through the release of pathogen- or damage-associated molecular patterns (PAMPs or DAMPs), thus influencing the efficacy of anti-tumor therapies (20–22). Therefore, exploring the relationship between macrophages and RCD has the potential to provide novel insights into cancer therapy and enhance therapeutic interventions.

This review aims to explore the intricate interactions between TAMs and RCD within the tumor microenvironment, and to analyze their potential roles in tumor progression and therapeutic intervention. By elucidating how TAMs and RCD mutually influence each other and their dual impact on tumor growth, this paper seeks to inspire innovative approaches in immuno-oncology and identify potential avenues for future research and clinical application.

## 2 Variability and categorizations in TAM

Macrophages are generally classified into two major phenotypes based on their function: classically activated M1 macrophages, which exhibit anti-tumor properties, and alternatively activated M2 macrophages, which promote tumor growth. M1 macrophages are characterized by their pro-inflammatory and anti-tumor activities, effectively recognizing and eliminating cancer cells through cytotoxic and phagocytic actions (23, 24). These cells secrete tumor necrosis factor-α (TNF-α), interleukins such as IL-1β, and nitric oxide (NO), and activate immune responses by presenting antigens via MHC-II molecules (25, 26). In contrast, M2 macrophages exhibit anti-inflammatory functions, promoting tissue repair and tumor progression. They secrete IL-4, IL-10, and transforming growth factor-β (TGF-β), which facilitate tumor growth and metastasis (7). M2 macrophages are also distinguished by surface markers such as CD206 and CD163, and they produce ECM components, further supporting tumor growth (27–29). Additionally, M2 macrophages release pro-angiogenic factors like vascular endothelial growth factor (VEGF), providing nutrients to tumors and promoting metastasis (30, 31). In the early stages of cancer, TAMs typically exhibit an M1 phenotype, activating anti-tumor immune responses and inhibiting angiogenesis. However, as the tumor progresses, TAMs gradually shift toward an M2 phenotype, promoting angiogenesis and further tumor development (32). Early-stage infiltration of M1 macrophages is often associated with increased IL-12 and decreased IL-10 levels, which enhances immune responses and cancer cell destruction. In later stages, TAMs exhibit M2-like characteristics, with reduced IL-12 and elevated IL-10 levels, impairing their anti-tumor capabilities (33). Overall, TAMs support tumor progression by promoting cancer cell survival, proliferation, angiogenesis, and metastasis (34, 35). Therefore, strategies aimed at reducing TAM infiltration, blocking specific signaling pathways, or disrupting their interactions with tumor cells may serve as effective cancer therapies (36).

**Figure 1:**
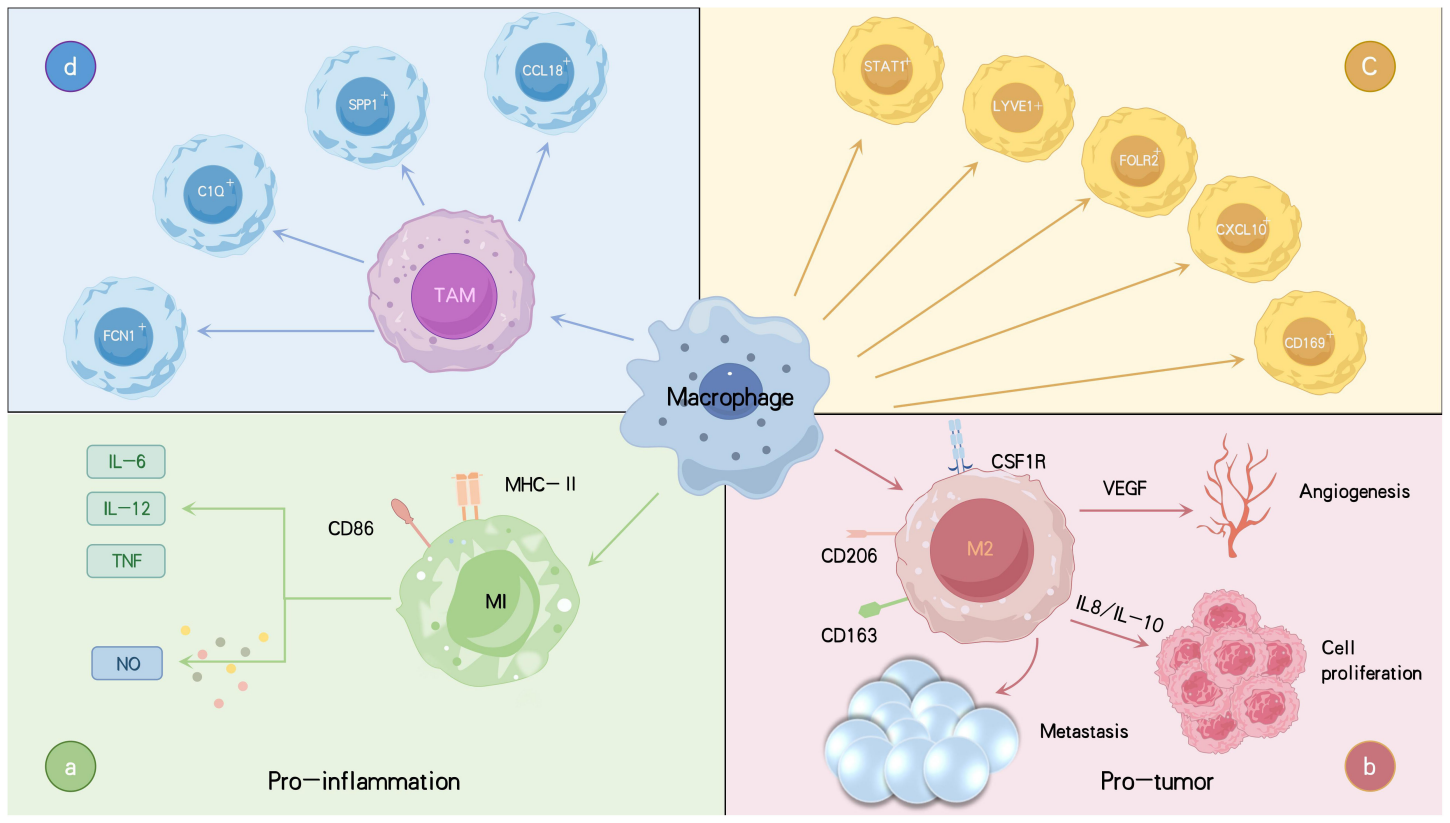
Varieties of macrophages linked to tumors along with their primary functions and roles. TAMs are categorized into two main phenotypes: the anti-tumor M1 type, which secretes pro-inflammatory cytokines such as IL-6, IL-12, and TNF-α, produces NO, and promotes inflammation **(A)**. The M2 type is pro-tumorigenic, secreting IL-8 and IL-10, which promote tumor progression and dissemination, as well as VEGF, which facilitates angiogenesis, thereby acting as a tumor promoter **(B)**. Macrophages are further classified into subsets CD169+, STAT1+, LYVE1+, FOLR2+ macrophages **(C)**. Recently, new TAM subtypes have been identified, including FCN1+, SPP1+, C1Q+, and CCL18+ TAMs, each with distinct functions in tumor progression and immune regulation **(D)**. Tumor necrosis factor (TNF), interleukins (IL), vascular endothelial growth factor (VEGF), nitric oxide (NO).

However, the distinction between M1 and M2 macrophages is often unclear, as these phenotypes overlap in both function and molecular expression, complicating their classification. While the traditional M1/M2 framework remains useful, it no longer fully captures the diversity and dynamic nature of macrophages in different microenvironments (37). Recent single-cell analyses have shown that M1 and M2 markers can be co-expressed within the same macrophage, highlighting the importance of identifying macrophage subpopulations beyond simple polarization (38). In recent years, several macrophage subtypes distinct from the traditional M1/M2 classification have been identified, including CD169+, STAT1+, LYVE1+, SPP1+, CXCL10+, and FOLR2+ macrophages, as well as regulatory macrophages (26, 39, 40). Single-cell RNA sequencing (scRNA-seq) has revealed novel TAM populations in solid tumors, such as FCN1^+^, SPP1^+^, C1Q^+^, and CCL18^+^ TAMs (41) (**Figure 1**). FCN1^+^ TAMs are less differentiated and primarily pro-inflammatory, contributing to early tumor-associated inflammation. As the TME evolves, FCN1^+^ TAMs may differentiate into other subtypes, such as C1Q^+^ TAMs (42). SPP1+ TAMs are recognized for their immunosuppressive and tumor-promoting effects, driving metastasis, angiogenesis, and cancer stem cell activation (42–44). C1Q^+^ TAMs secrete immunosuppressive molecules such as IL-10 and TGF-β, inducing Treg cell differentiation and suppressing effector T cells (45–47). They also retain some antigen-presenting and phagocytic capabilities, allowing them to serve multiple functions in immune regulation. CCL18+ TAMs represent terminally differentiated, immunosuppressive macrophages with typical M2-like characteristics. They promote tumor cell proliferation, migration, invasion, epithelial-mesenchymal transition (EMT), angiogenesis, and lymphangiogenesis, contributing to the malignant progression of tumors. These TAM subpopulations undergo complex signaling regulation and can interconvert at different stages of tumor progression (48). Monocyte-derived macrophages can differentiate into FCN1+ and C1Q+ TAMs, while embryonic tissue-resident macrophages (TRMs) primarily give rise to SPP1+ and CCL18+ TAMs (41). Although these newly discovered TAM subpopulations differ from the traditional M1/M2 classification, they exhibit greater functional flexibility, demonstrating continuity and dynamic changes regulated by the complex tumor microenvironment. Compared to the conventional M1/M2 paradigm, these subtypes provide a more nuanced understanding of macrophage diversity and plasticity in tumors.

## 3 Types of RCD and their interactions with TAMs

### 3.1 Apoptosis

**Figure 2:**
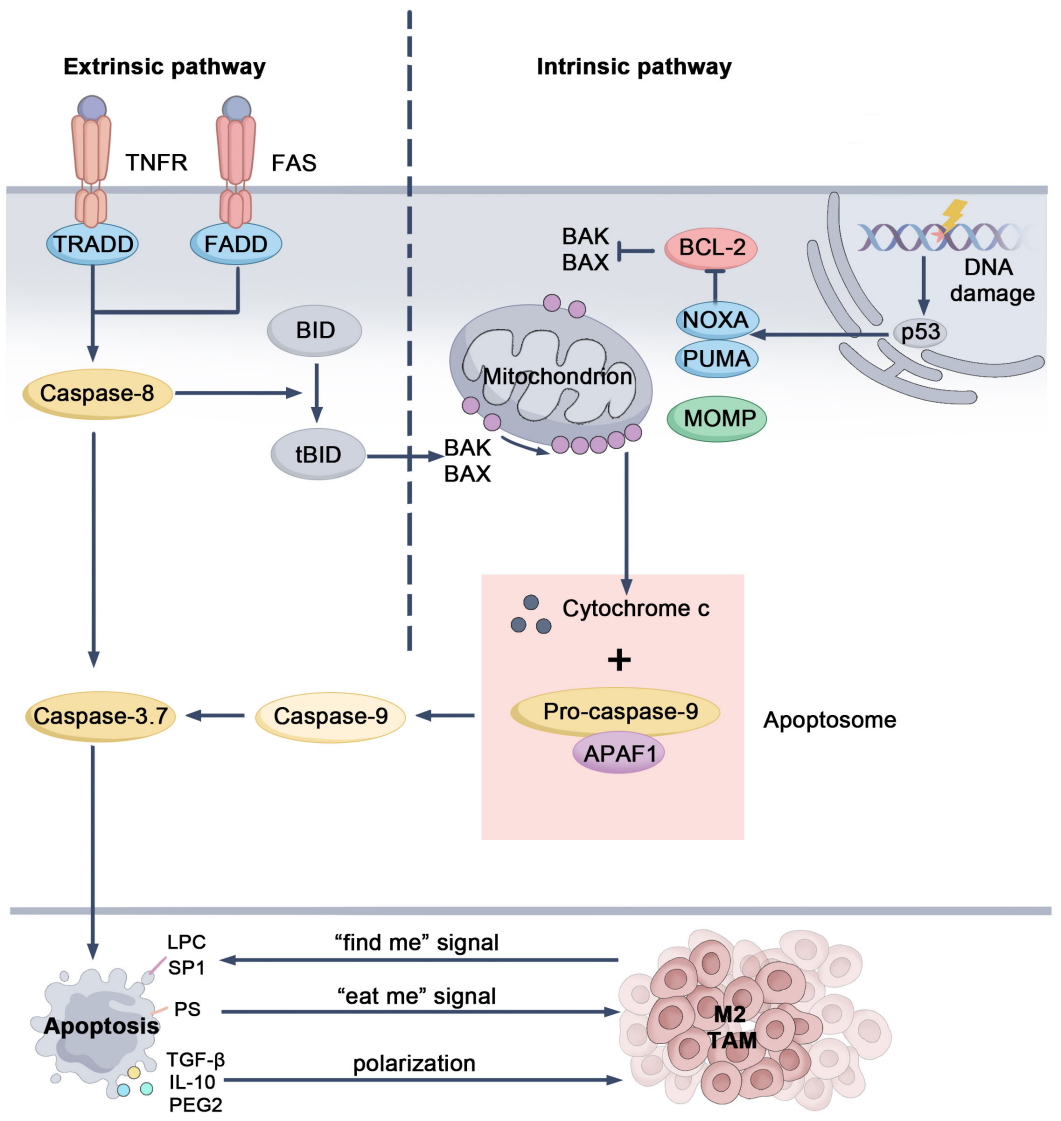
Mechanistic pathways and regulatory targets of apoptosis. When DNA is damaged, the tumor suppressor protein p53 is activated, triggering the expression of pro-apoptotic genes like *Noxa* and *Puma*. These proteins inhibit the anti-apoptotic Bcl-2 family members, allowing Bax and Bak to oligomerize on the mitochondrial outer membrane. This causes MOMP, leading to the release of cytochrome c. Once released, cytochrome c combines with APAF-1 and pro-caspase-9, forming the apoptosome. The apoptosome activates caspase-9, which subsequently activates caspase-3 and caspase-7, ultimately driving the cell through apoptosis. In the extrinsic pathway, signals from death receptors trigger the activation of caspase-8. Caspase-8 can directly activate effector caspases or cleave BID into tBID, which then interacts with Bax and Bak, connecting this pathway to the intrinsic one. During apoptosis, tumor cells release “find-me” signals to recruit macrophages and express “eat-me” signals to facilitate phagocytosis. They also release immunosuppressive molecules that can shift macrophages towards an immunosuppressive TAM phenotype, contributing to the tumor’s immune evasion. BH3-interacting domain death agonist (BID), Bcl-2-associated X protein (Bax), Bcl-2 killer (Bak), Mitochondrial Outer Membrane Permeabilization (MOMP), Apoptotic protease activating factor 1 (APAF-1).

Apoptosis, a form of programmed cell death, is a tightly regulated process that maintains tissue homeostasis by eliminating damaged or abnormal cells, thereby preventing mutation accumulation and inhibiting tumor growth during early tumorigenesis (49). Apoptosis is initiated through two main pathways: the intrinsic (mitochondrial) and extrinsic (death receptor) pathways. The intrinsic pathway is initiated by intracellular stressors, including DNA damage and reactive oxygen species (ROS). These signals activate pro-apoptotic proteins BAX and BAK, which permeabilize the mitochondrial outer membrane, releasing cytochrome c. Cytochrome c combines with APAF-1 and pro-caspase-9 to form the apoptosome, activating caspase-9 and initiating a cascade that activates effector caspases like caspase-3, leading to cell death (50, 51). The extrinsic pathway is activated by external signals, such as FasL and TNF, which bind to death receptors on the cell surface, leading to the activation of caspase-8. This pathway can interact with the intrinsic pathway to promote apoptosis (50, 52) (**Figure 2**). Both pathways converge at the execution phase, where caspases degrade intracellular structures, resulting in cell death. Unlike other forms of cell death, apoptosis is considered a “silent” form of death, as it does not provoke a prominent inflammatory response. Following cell death, phagocytes, including macrophages, efficiently clear residual cellular debris, ensuring tissue homeostasis and immune balance (53).

In the TME, the interaction between apoptotic tumor cells and TAMs significantly influences tumor progression (54, 55). Apoptotic cells release “find-me signals,” such as lysophosphatidylcholine (LPC) and sphingosine-1-phosphate (S1P), which attract TAMs to the site of cell death. Furthermore, interactions with “eat-me signals,” such as phosphatidylserine (PS), promote the reprogramming of TAMs to an M2-like phenotype (51, 56). This interaction drives TAMs to secrete anti-inflammatory cytokines, such as TGF-β and IL-10, as well as pro-tumor factors like VEGF and EGF, thereby supporting tumor growth, promoting angiogenesis, and suppressing immune responses. Additionally, S1P induces TAMs to secrete prostaglandin E2 (PGE2), which further inhibits cytotoxic T cell activity and contributes to tumor recurrence (51, 57–59). In therapeutic settings, treatments like radiotherapy and chemotherapy aim to inhibit tumor growth by inducing apoptosis in tumor cells. However, research suggests that the signals released by apoptotic cells can activate the anti-inflammatory and tissue-repairing functions of TAMs, potentially promoting tumor recurrence and prolonged growth (51). Furthermore, TAMs can influence the rate of apoptosis in tumor cells. For example, cytokines such as IL-6 and IL-10, secreted by TAMs to aid tissue repair, may inhibit cancer cell apoptosis by activating survival signaling pathways, including signal transducer and activator of transcription 3 (STAT3) and nuclear factor kappa B (NF-κB) (60–62).

Thus, targeting the interactions between TAMs and apoptotic cells represents an effective cancer therapeutic strategy. Nevertheless, many cancer cells suppress apoptosis by downregulating the expression of caspase-8 and overexpressing anti-apoptotic proteins, limiting the potential for apoptosis-mediated tumor cell death (50). In these scenarios, targeting TAMs in conjunction with inducing alternative cell death pathways, such as necroptosis or ferroptosis, may be a crucial approach to overcoming cancer cell apoptosis resistance.

### 3.2 Necroptosis

**Figure 3:**
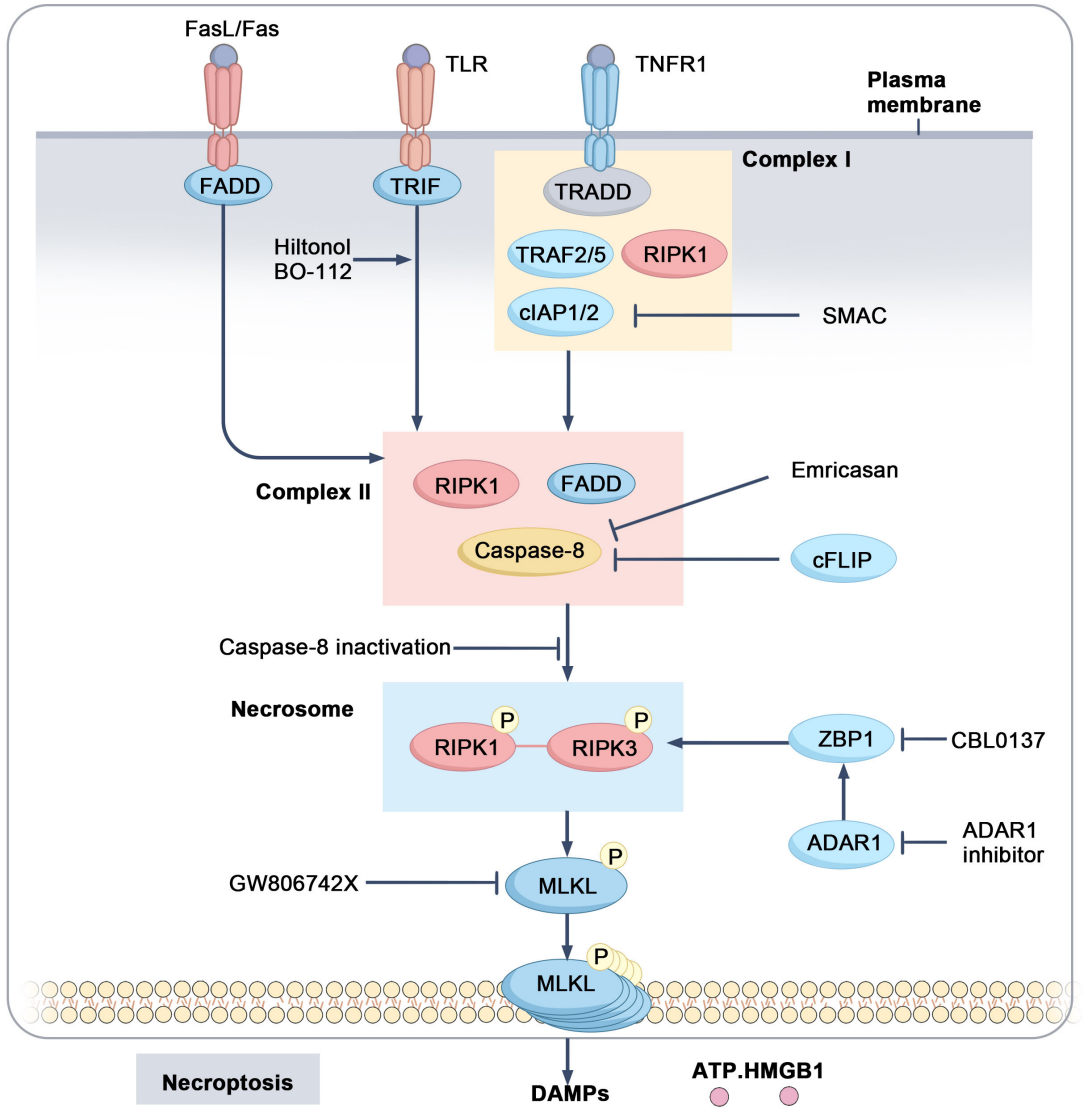
Mechanistic pathways and regulatory targets of necroptosis. Upon activation of TNFR1, a complex I is formed consisting of TRADD, TRAF2/5, cIAP1/2, and RIPK1. cIAP1/2 ubiquitinates RIPK1, thereby inhibiting its activation. When caspase-8 and IAP activity are suppressed, RIPK1 is activated and forms complex II with FADD and caspase-8, which further activates RIPK3. The activated RIPK1-RIPK3 complex forms the necrosome, leading to the activation of MLKL and inducing necroptosis, accompanied by the release of DAMPs such as ATP and HMGB1.

Necroptosis is a programmed form of cell death that depends on receptor-interacting protein kinase 1 (RIPK1) and receptor-interacting protein kinase 3 (RIPK3), which activate mixed lineage kinase domain-like protein (MLKL) through phosphorylation. This process is typically initiated by death receptors such as TNFR and Fas or Toll-like receptors 3/4 (TLR3/4) (63–67). When TNFR is activated, it forms a complex comprising RIPK1, Fas-associated death domain (FADD), TNFR-associated death domain (TRADD), TNFR-associated factors (TRAFs), and cellular inhibitors of apoptosis proteins (cIAP1/2). Within this complex, cIAP1/2 ubiquitinates RIPK1, preventing the formation of the RIPK1-RIPK3 complex and promoting cell survival by inhibiting apoptosis (68, 69). When cIAP1/2 is inhibited, RIPK1 is recruited to an oligomeric complex formed by FADD and caspase-8. Cellular FLICE-inhibitory protein (cFLIP), which inhibits caspase activity, is often upregulated in tumors, helping cells evade death signals (70). If caspase-8 function is compromised (e.g., due to upregulation of cFLIP), RIPK1 further recruits and activates RIPK3, resulting in the formation of a “necrosome.” Subsequently, RIPK3 phosphorylates MLKL, which translocates to the cell membrane, ultimately leading to membrane rupture and the chaotic release of cellular contents (63, 64, 71, 72) (**Figure 3**). Similar to other forms of regulated cell death, such as ferroptosis, necroptosis plays a crucial role in tumor initiation, progression, death, and immune response.

The interaction between necroptosis and TAMs is intricate. The necroptotic signaling pathway mediated by RIPK1, RIPK3, and MLKL regulates the function of TAMs, influencing tumor progression. For example, in pancreatic cancer, upregulation of RIPK1 inhibits STAT1 activity in macrophages, promoting M2 polarization and immune evasion. Inhibition of RIPK1 expression can activate cytotoxic T cells and drive macrophage polarization toward the M1 phenotype, thereby enhancing the efficacy of immunotherapy (73). In hepatocellular carcinoma (HCC), downregulation of RIPK3 induces fatty acid oxidation within the TME, facilitating M2 polarization of TAMs and subsequently driving tumor initiation and progression (74). In pancreatic ductal adenocarcinoma (PDAC), MLKL has been observed to enhance CD47-SIRPα signaling in tumor cells, which in turn inhibits macrophage phagocytosis and enables tumor cells to evade immune clearance. Additionally, CXCL8 released during necroptosis induces macrophages to form extracellular traps (METs), which promote extracellular matrix degradation and metastasis (75). Necroptosis also recruits myeloid-derived suppressor cells (MDSCs) through the release of chemokines such as CXCL1 and CXCL5, contributing to an immunosuppressive TME that facilitates tumor migration and invasion (76, 77). Despite the immunostimulatory properties of necroptosis-released substances like ATP and High Mobility Group Box 1 (HMGB1), which support the differentiation of M1 macrophages (49), the TME involved in necroptosis tends to favor immune suppression (78, 79). Research on the role of TAMs in regulating necroptosis remains limited and urgently requires further exploration.

### 3.3 Pyroptosis

Pyroptosis is an inflammatory form of cell death characterized by cell swelling and membrane pore formation, distinguishing it from apoptosis. Central to pyroptosis are the Gasdermin family of proteins, particularly Gasdermin D (GSDMD) and GSDME. Upon cleavage, the N-terminal domain of these proteins forms pores in the plasma and mitochondrial membranes, leading to cell swelling and the release of cytokines, which triggers a strong inflammatory response​ (80, 81). Pyroptosis is primarily regulated through two main pathways: the canonical and non-canonical pathways. The canonical pathway is activated in response to PAMPs or DAMPs. In this context, inflammasomes such as NLRP3 are activated, which in turn activate caspase-1. Activated caspase-1 cleaves GSDMD, resulting in pore formation in the tumor cell membrane, cell lysis, and the induction of pyroptosis. Concurrently, caspase-1 cleaves pro-IL-1β and pro-IL-18, releasing their mature forms to amplify the inflammatory response within the TME (82, 83). The non-canonical pathway is mediated by caspase-4, caspase-5 (in humans), or caspase-11 (in mice), which are typically activated by stimuli like lipopolysaccharides (LPS). These caspases directly cleave GSDMD, leading to pyroptosis. This inflammatory response enhances the anti-tumor effects of the immune system and may inhibit tumor growth and dissemination by modulating T cells and NK cells (84–86). However, excessive activation of pyroptosis may support tumor cell survival and metastasis, negatively impacting tumor immunity (84).

**Figure 4:**
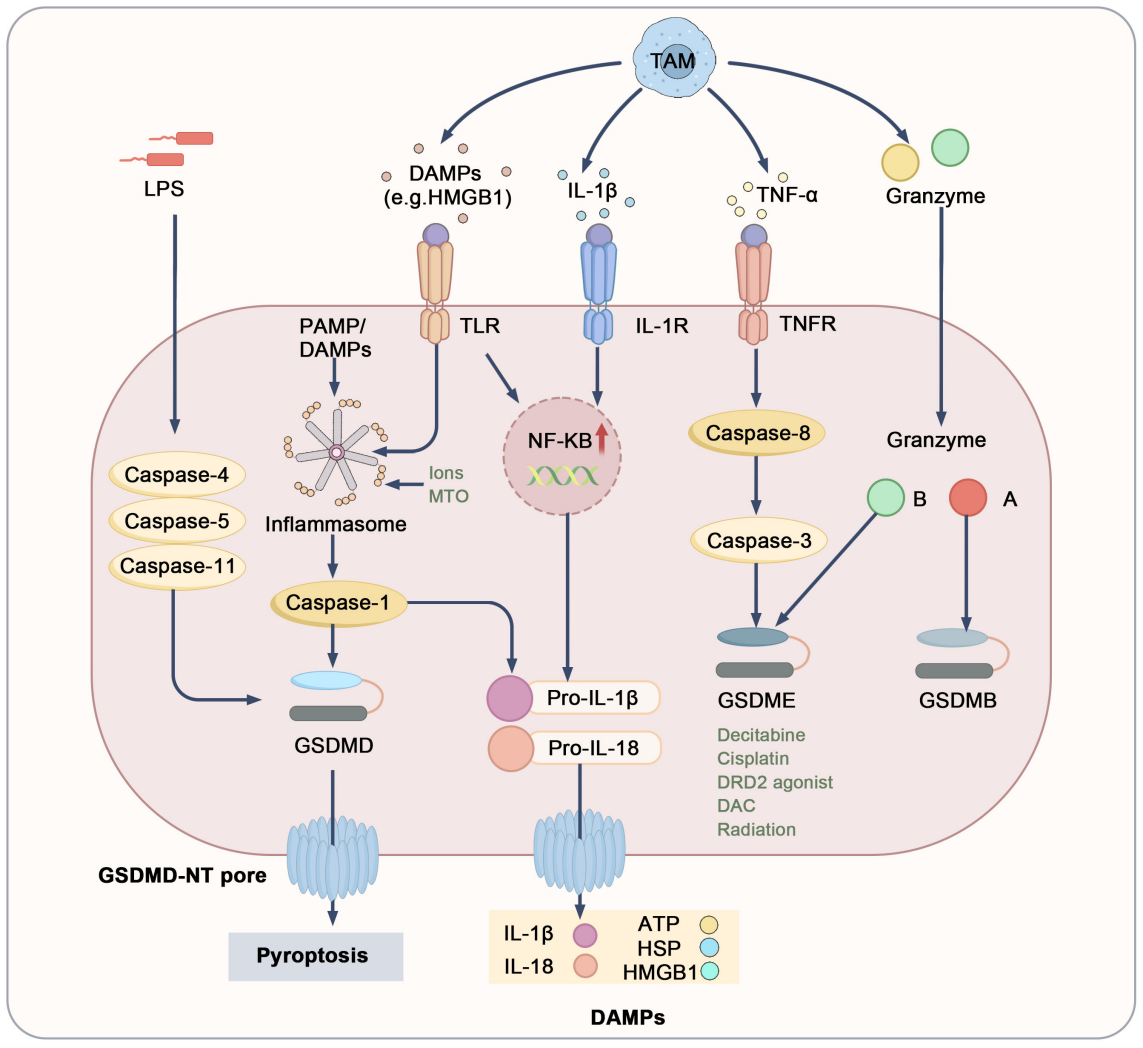
Mechanistic pathways and regulatory targets of pyroptosis. DAMPs, cytokines, and granzymes derived from TAMs or other cells interact with surface receptors, including TLRs, IL-1R, and TNFR1, or directly induce pyroptosis. The execution of pyroptosis relies on the cleavage of gasdermin proteins, leading to pore formation, the release of mature IL-1β and IL-18, and the leakage of cellular contents, which results in the release of DAMPs including ATP, HSPs, and HMGB1. Toll-like receptors (TLRs), heat shock proteins (HSPs), IL-1 receptor (IL-1R).

The interaction between pyroptosis and TAMs plays a crucial role in regulating the tumor microenvironment (**Figure 4**). Specifically, TAMs induce pyroptosis in tumor cells by secreting pro-inflammatory factors, DAMPs, and granzymes (84, 87–89). Among these pro-inflammatory factors, cytokines such as TNF-α and IL-1β are particularly significant, as they activate downstream signaling pathways like NF-κB, promoting the formation of inflammasomes and resulting in the production of pro-IL-1β and pro-IL-18 (90–93). TNF-α secreted by TAMs also recruits caspase-8 through a complex pathway, leading to GSDME cleavage by caspase-3 (98). Furthermore, various stimuli encourage TAMs to release pro-inflammatory mediators like HMGB1, further contributing to inflammasome activation (94). TAMs also express high levels of granzymes and perforin, which can directly enter target tumor cells. Notably, granzyme B cleaves GSDME, while granzyme A targets GSDMB (95–97). Additionally, pyroptosis may facilitate immune cell infiltration into the TME, promoting M1 macrophage recruitment and facilitating anti-tumor responses. The rupture of tumor cells undergoing pyroptosis releases a number of inflammatory factors, including IL-18, IL-1β, HSP, ATP, and HMGB1. These molecules are recognized and engulfed by TAMs, prompting their differentiation into M1 macrophages, which further induce pyroptosis in tumor cells and activate additional immune cells, creating a positive feedback loop that improves the TME (49, 98–100). However, chronic inflammation induced by sustained pyroptosis may drive TAMs to polarize into M2 macrophages, typically associated with tumor promotion. Elevated levels of IL-1β have been linked to tumor invasion and metastasis (101). Notably, NLRP3-driven pyroptosis and IL-1β release from metastatic sites can induce the expression of various chemokines, such as CCL5, CXCL12, CCL2, and CXCL5. These chemokines attract MDSCs and M2 macrophages, enhancing immune suppression and promoting tumor growth and metastasis (102, 103). In summary, the dual role of TAMs in pyroptosis ultimately influences tumor progression. Nevertheless, pyroptosis continues to be a promising target for cancer therapy; precise modulation of pyroptotic pathways and TAM polarization may offer new strategies for tumor immunotherapy.

### 3.4 Ferroptosis

**Figure 5:**
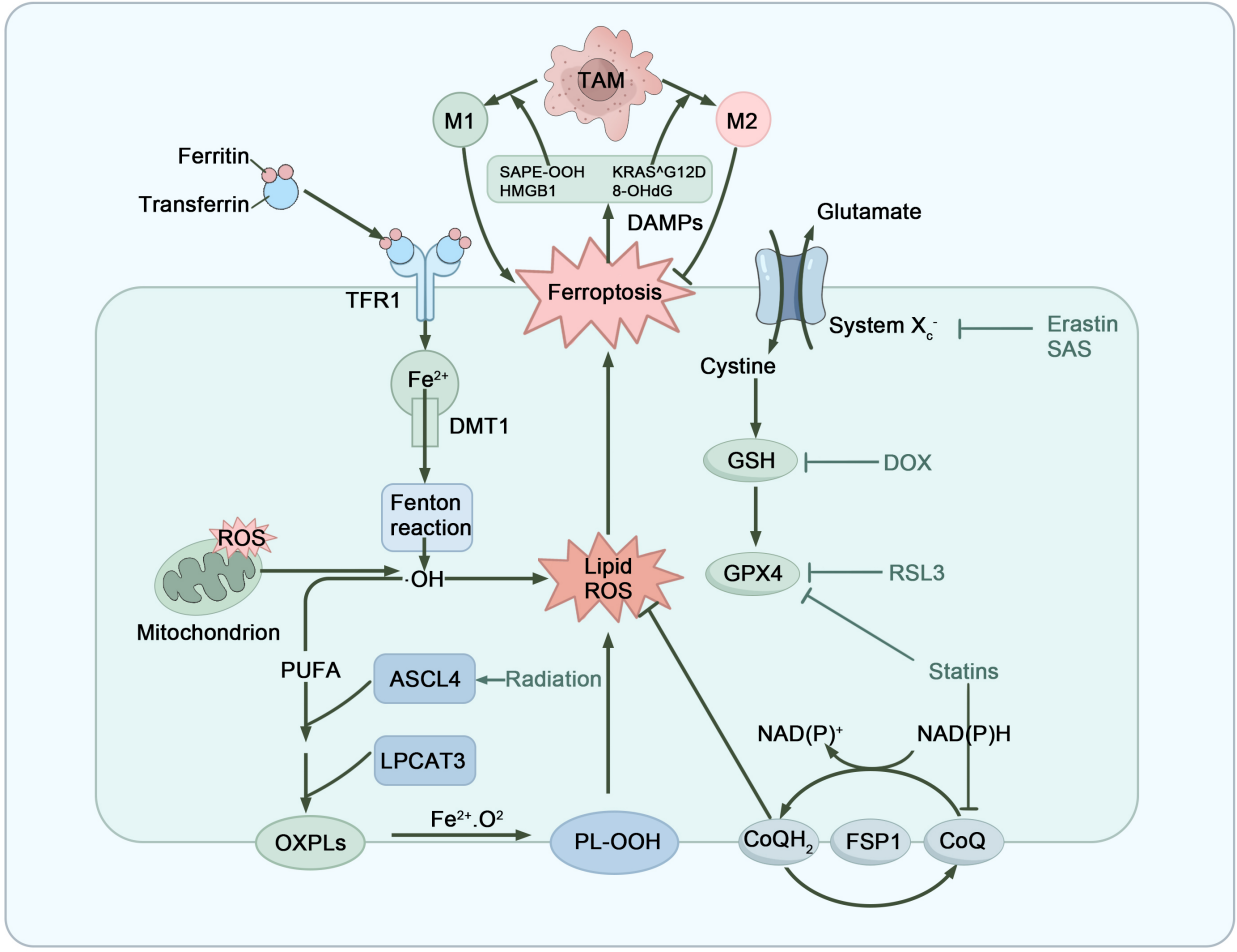
Mechanistic pathways and regulatory targets of ferroptosis. Ferroptosis is a form of cell death induced by iron overload and the accumulation of lipid peroxides. The Fenton reaction generates hydroxyl radicals via the interaction of Fe^2+^ and H_2_O_2_, which subsequently react with lipids to create lipid radicals, initiating lipid peroxidation. When antioxidant defenses, including the Xc^−^ system/GSH/GPX4 and FSP1/CoQ10/NAD(P)H pathways are suppressed, cells lose the ability to effectively inhibit lipid peroxidation, resulting in excessive ROS generation and ultimately causing cell death. DAMPs released from dying cells, including SAPE-OOH, HMGB1, KRAS^G12D mutant protein, and 8-hydroxy-2’-deoxyguanosine, are capable of regulating TAM differentiation. Meanwhile, M1-type TAMs promote ferroptosis in tumor cells, whereas M2-type TAMs exert inhibitory effects. Oxidized Phospholipids (OXPLs), Phospholipid Hydroperoxides (PL-OOH).

Ferroptosis is an iron-dependent form of programmed cell death, primarily triggered by intracellular iron accumulation and excessive lipid peroxidation. The mechanism involves the Fenton reaction of hydrogen peroxide with free iron to generate hydroxyl radicals, which leads to the peroxidation of polyunsaturated fatty acids and the production of lipid reactive oxygen species (lipid ROS). This disrupts cellular membrane integrity, ultimately resulting in cell death (104). Phospholipid hydroperoxide glutathione peroxidase 4 (GPX4) and its cofactor glutathione (GSH) play critical roles in inhibiting lipid peroxidation, constituting a major cellular defense mechanism against ferroptosis. Additionally, ferroptosis suppressor protein 1 (FSP1) enhances cellular antioxidant capacity through the regeneration of coenzyme Q10 (CoQ10), thereby mitigating the effects of lipid peroxidation (105, 106). However, when the system Xc^−^ (cystine-glutamate transporter)/GSH/GPX4 pathway and the FSP1/CoQ10/NAD(P)H pathway are inhibited, lipid peroxide accumulation damages both plasma and mitochondrial membranes, ultimately triggering cell death (107, 108) (**Figure 5**).

Ferroptosis not only directly kills tumor cells but also influences tumor progression by regulating macrophage function and polarization. Acting as a double-edged sword, ferroptosis can enhance the anti-tumor effects of M1 macrophages while also impacting the immunosuppressive functions of M2 macrophages (109). DAMPs released during ferroptosis interact with pattern recognition receptors (PRRs) on macrophages to regulate their polarization. For instance, 1-stearoyl-2-15-hydroxyeicosatetraenoic acid phosphatidylethanolamine (SAPE-OOH) and HMGB1 are released during tumor ferroptosis and bind to TLR2 and RAGE receptors. These signals can activate and recruit immune cells to the tumor site, thereby enhancing anti-tumor immune responses (110–112)​. Conversely, specific DAMPs, such as the KRAS^G12D mutant protein and 8-hydroxy-2’-deoxyguanosine (8-OHdG), bind to AGER and TMEM173 receptors, promoting the polarization of M2 macrophages and inhibiting anti-tumor immune responses (113, 114). Furthermore, ferroptosis in the tumor microenvironment is often accompanied by abnormal iron metabolism, which affects the differentiation of TAMs. Studies indicate that tumor cells competitively inhibit transferrin receptor 1 (TFR1), limiting iron supply and modulating macrophage energy metabolism, promoting M2 polarization while decreasing their sensitivity to ferroptosis (115).

On the other hand, TAMs are also instrumental in regulating ferroptosis (**Figure 5**). M1 macrophages enhance ferroptosis by activating CD8+ T cells and releasing IFN-γ, which downregulates the expression of the cystine-glutamate antiporter system SLC3A2 and SLC7A11, resulting in reduced GSH production and impaired GPX4 function. Additionally, the activation of ACSL4 (Acyl-CoA Synthetase Long-Chain Family Member 4) by M1 macrophages increases the oxidative sensitivity of tumor cell membrane phospholipids, thereby promoting ferroptosis (116–118). Furthermore, M1 macrophages release peroxides that accelerate the Fenton reaction, generating ROS and further inducing lipid peroxidation and cell membrane damage (119, 120). In contrast, M2 macrophages indirectly inhibit ferroptosis through various mechanisms, enhancing the survival of tumor cells. They achieve this by inhibiting CD8+ T cell activation, upregulating PD-L1 expression in tumor cells, secreting TGF-β1, and interfering with Arachidonate 15-Lipoxygenase (ALOX15) expression, thereby promoting tumor progression (121–124). Recent studies in mouse models of laryngeal squamous cell carcinoma have shown that exosomes secreted by M2 macrophages, rich in annexin A3 (ANXA3), enhance the synthesis of GSH and promote intracellular iron accumulation, thereby increasing tumor cell resistance to ferroptosis (125). Additionally, taurine secreted by M2 macrophages promotes lipid synthesis in tumor cells by activating the Liver X receptor α (LXRα) and Stearoyl-CoA desaturase 1 (SCD1) pathways, which reduces intracellular lipid peroxidation levels and effectively inhibits ferroptosis in liver cancer cells (126). Concurrently, Schwantes et al. found that under the regulation of hypoxia-inducible factor 2 (HIF-2) and STAT1, TAMs significantly increase the expression of ceruloplasmin in tumor cells through exosomes, further enhancing tumor cell resistance to RAS-selective lethal 3 (RSL3)-induced ferroptosis (127). In summary, the interactions between TAMs and ferroptosis reveal the potential for enhancing ferroptosis in tumor immunotherapy through the modulation of macrophage polarization, providing a theoretical basis for developing new cancer immunotherapy strategies.

## 4 Therapeutic implications in immuno-oncology

### 4.1 Therapeutic strategies inducing RCD subtypes and their impact on TAM

#### 4.1.1 Induction of pyroptosis

In recent years, pyroptosis has emerged as a significant research focus in cancer therapy, particularly in conjunction with advanced treatment strategies such as nanotechnology, chemotherapy, and immune checkpoint inhibitors. Research has demonstrated that the pyroptosis pathway not only induces cancer cell death but also stimulates robust immune responses by releasing tumor-associated antigens (TAAs) and pro-inflammatory factors, which notably affect TAMs (128–130).

Decitabine, a DNA demethylating agent that inhibits DNA methyltransferases, has been shown to demethylate the DFNA5 gene, thereby restoring GSDME expression. In breast cancer and colon cancer, the combination of Decitabine with the chemotherapeutic agent cisplatin activates the Caspase-3-GSDME pathway, leading to pyroptosis (131, 132). Additionally, DRD2 expression in breast tumor cells has been found to induce pyroptosis via activation of the NOD-like Receptor Protein 3 (NLRP3) inflammasome and Caspase-1, releasing pro-inflammatory factors that recruit and activate M1 macrophages and natural killer (NK) cells (100). This process creates a positive feedback loop, promoting M1 macrophage polarization and amplifying the immune response (100, 129, 132). As a result, DRD2 is emerging as a potential therapeutic target, and dopamine receptor agonists are being explored for their ability to promote DRD2 activation in cancer therapy (100).

Recent advances in drug delivery systems, such as nanoparticles and hydrogels, have opened new possibilities for cancer treatment by improving tumor-targeting precision and minimizing toxicity to normal tissues (133). For example, zinc-phenol nanocapsules (RMP@Cap) contain zinc ions, mitoxantrone (MTO), and anti-PD-L1 antibodies, and induce pyroptosis through the activation of the NLRP3-Caspase-1-GSDMD axis (134). This process triggers mitochondrial membrane remodeling and mtDNA release, which activates the cGAS-STING pathway, further enhancing the tumor immune response (135). Encapsulation with red blood cell membranes extends *in vivo* circulation of the nanocapsules, increasing drug accumulation at the tumor site and enhancing therapeutic efficacy (134). Likewise, metal-organic framework-based nanovaccines (Cu-THBQ/AX) induce pyroptosis and promote M1 macrophage polarization by generating ROS and activating the PLC-Ca^2+^-Caspase-3-GSDME pathway, thus boosting anti-tumor immunity (136). Demethylating agents like Decitabine (DAC) has also been shown to upregulate GSDME expression in breast cancer (137). A novel biohybrid system developed by Xing et al. employs macrophage-based microbots (IDN@MC) to deliver the photosensitizer IR and DAC via macrophages. Upon laser irradiation, the microbots trigger photothermal effects and activate the Caspase-3-GSDME pathway, inducing pyroptosis. Macrophages in this system not only serve as drug carriers but also polarize toward the M1 phenotype, enhancing the anti-tumor immune response​ (138).

Bacterial therapy has also shown significant potential in activating pyroptosis. Zhang et al. targeted glioblastoma (GBM) using genetically modified Salmonella, whose lysis releases LPS that binds to TLR4 on host cells, activating the NLRP3-Caspase-1-GSDMD pathway and inducing pyroptosis. This therapy effectively promotes M1 macrophage polarization and significantly reduces postoperative GBM recurrence (139). Additionally, Yin et al. developed an ultrasound-controlled perforation system (UPS) that activates the Caspase-3-GSDME pathway and induces pyroptosis while restoring GSDME expression, significantly enhancing the efficacy of radioimmunotherapy (140).

Although pyroptosis holds great promise for tumor immunotherapy, its clinical application faces several critical challenges. One of the major hurdles is to effectively induce pyroptosis in cancer cells while minimizing unwanted side effects. Cancer cells often evade pyroptosis by modifying or downregulating crucial proteins involved in the pyroptosis pathway, such as GSDME (141). Moreover, the intense inflammatory response and non-specific effects of pyroptosis can extend beyond tumor cells, potentially harming surrounding healthy tissues. In clinical contexts, prolonged or uncontrolled inflammation may limit the therapeutic window and lead to severe complications, including cytokine release syndrome (CRS) (142). To overcome these obstacles, controlling the inflammatory response, either through the use of anti-inflammatory agents or by precisely regulating pyroptosis activation, could help retain its therapeutic potential while minimizing adverse effects. For example, inhibitors of the NLRP3 inflammasome, such as ZYIL1, have entered clinical trials to reduce excessive inflammatory responses caused by inflammasome activation (143). Additionally, future research should focus on enhancing tumor cell sensitivity to pyroptosis and exploring whether combining pyroptosis with chemotherapy or immunotherapy could improve therapeutic outcomes (69).

#### 4.1.2 Induction of ferroptosis

Ferroptosis inducers encompass a wide range of agents, including targeted therapies, chemotherapy, lipid-lowering agents, immune therapies, and radiotherapy, all of which have shown the ability to induce ferroptosis and suppress tumor growth. Type I ferroptosis inducers reduce cysteine uptake by inhibiting the system Xc^−^, with Erastin being a representative example. Erastin primarily inhibits SLC7A11, a crucial component of system Xc^−^ (144). Studies indicate that Erastin significantly enhances anti-cancer effects, particularly in RAS-mutant cancer cells, although its clinical application remains limited (118). Additionally, sorafenib, commonly used in HCC, can also induce ferroptosis by targeting the SLC7A11 subunit (144). Jiang et al. developed a composite of sulfamethazine (SAS) encapsulated in magnetic nanoparticles (Fe_3_O_4_) and coated with platelet membranes (Fe_3_O_4_-SAS@PLT). This composite inhibits system Xc^−^ via SAS, while Fe^2+^ released from Fe_3_O_4_ triggers the Fenton reaction, inducing ferroptosis. Experiments reveal that this approach not only induces ferroptosis but also polarizes macrophages toward the M1 phenotype, enhancing their response to PD-1 therapy (145).

Type II ferroptosis inducers directly target and inactivate GPX4, thereby disrupting the cellular antioxidant defense. RSL3, a representative drug of this class, binds to GPX4 and inhibits its activity, preventing the clearance of intracellular lipid peroxides. RSL3 effectively induces ferroptosis in cancer cells that are highly dependent on antioxidant mechanisms (146–148). Additionally, RSL3 has also been shown to promote the polarization of M1 macrophages (149). Doxorubicin (DOX), a widely used chemotherapeutic agent, inhibits tumor cell proliferation and promotes apoptosis by interfering with DNA synthesis (150). A gold nanocage platform (m@Au-D/B NCs) loaded with DOX and L-buthionine sulfoximine (BSO) has been designed to deplete GSH, accumulate ROS, and induce ferroptosis. This platform also shifts TAMs from the M2 to M1 phenotype through combined photothermal therapy (PTT) and ROS, thereby improving the tumor microenvironment (151).

Type III ferroptosis inducers deplete intracellular GPX4 and CoQ10, an antioxidant that prevents ROS accumulation and lipid peroxidation. Statins, commonly used in clinical settings, reduce CoQ10 synthesis by inhibiting HMG-CoA reductase (152). For instance, Lovastatin not only induces ferroptosis but also downregulates PD-L1 expression in lung cancer cells, converting “cold” tumors into “hot” tumors and increasing their sensitivity to immunotherapy (153). Additionally, enzymes such as ACSL4 and Lysophosphatidylcholine Acyltransferase 3 (LPCAT3) are involved in phospholipid oxidation during ferroptosis, and their inhibitors may offer new therapeutic strategies. Research suggests that PKCβII senses initial lipid peroxidation and activates ACSL4 via phosphorylation, amplifying lipid peroxidation associated with ferroptosis (154). Radiotherapy has also been shown to induce ACSL4 expression, promoting lipid peroxidation and triggering ferroptosis (155). Moreover, Fatty Acid-Binding Protein 5 (FABP5), which regulates fatty acid transport and metabolism, is significantly elevated in liver cancer cells, protecting them from ferroptosis. Inhibiting FABP5 has been shown to increase intracellular polyunsaturated fatty acids (PUFAs) and ACSL4, leading to enhanced lipid ROS accumulation, ferroptosis induction, and M1 macrophage polarization (156). Therefore, targeting the PKCβII-ACSL4 and FABP5 pathways may represent a promising research objective.

The combination of ferroptosis with immunotherapy demonstrates significant therapeutic potential. As previously mentioned, CD8^+^ T cells, activated through immunotherapy, promote ferroptosis in tumor cells by secreting IFN-γ, which inhibits system Xc^−^ and boosts ACSL4 expression (157). Xu et al. developed a dual-function nanoplatform (SRF@Hb-Ce6) that carries sorafenib. This platform combines hemoglobin with the photosensitizer chlorin e6 (Ce6), supplying oxygen for oxygen-dependent photodynamic therapy (PDT) and providing iron for iron-dependent ferroptosis. In addition to inducing ferroptosis, PDT recruits immune cells, prompting them to secrete IFN-γ, which further enhances the effects of ferroptosis. These studies have shown promising results in both laboratory and animal models (158). The combination of immune checkpoint inhibitors (ICIs) and ferroptosis inducers has also demonstrated synergistic potential in suppressing tumor growth. For instance, in the LAR subtype of triple-negative breast cancer (TNBC), combining GPX4 inhibitors with ICIs, such as anti-PD-1, has proven more effective compared to monotherapy (159). However, given that ferroptosis may trigger immune suppressive activities, such as upregulation of PD-L1 and infiltration of MDSCs, further research is needed to effectively combine ferroptosis inducers with immunotherapy to mitigate immune suppression (121).

#### 4.1.3 Induction of necroptosis

The decision of whether to inhibit or promote necroptosis for cancer therapy is complex and highly dependent on the tumor type and treatment stage. Current research suggests that chronic or spontaneous necroptosis in certain cancers may dampen anti-tumor immunity, thereby facilitating tumor progression (160). Conversely, acute, large-scale necroptosis, triggered by treatments such as chemotherapy or radiotherapy, has the potential to inhibit tumor growth and elicit strong immunogenic responses. Thus, carefully regulating necroptosis, particularly in advanced cancer stages, may offer a promising anti-metastatic treatment strategy (161).

One method to exploit necroptosis for tumor inhibition involves combining Second Mitochondria-Derived Activator of Caspases (SMAC) mimetics with caspase inhibitors (162, 163). SMAC mimetics degrade IAPs, releasing the ubiquitination suppression on RIPK1, thereby promoting RIPK1-RIPK3 interaction and triggering necroptosis (164, 165). Concurrently, caspase inhibitors like Emricasan block Caspase-8, favoring necroptosis over apoptosis. In acute myeloid leukemia models, this combination therapy significantly promoted necroptosis and improved survival in mice (166). SMAC mimetics such as Debio 1143 and Birinapant, when used alongside Emricasan, have shown potential therapeutic effects in both preclinical and clinical trials (164, 165).

Another strategy involves directly activating RIPK3 using Z-DNA Binding Protein 1 (ZBP1) agonists, initiating necroptosis independently of RIPK1. In melanoma models resistant to immune checkpoint inhibitors, the ZBP1 agonist CBL0137 effectively enhanced necroptosis and reversed this resistance (167). Since Adenosine Deaminase Acting on RNA 1 (ADAR1), an RNA editing enzyme, inhibits ZBP1 activation, combining ADAR1 inhibitors with ZBP1 agonists holds promise for tumors unresponsive to immune checkpoint inhibitors. However, clinically available ADAR1 inhibitors are still under development (167, 168). Additionally, Toll-like receptor (TLR) agonists can induce necroptosis by activating the TRIF-RIPK3 signaling pathway. TLR3 agonists, such as Hiltonol and BO-112, may exhibit synergistic potential when combined with anti-PD-1 immunotherapy, amplifying the interplay between necroptosis and immune checkpoint inhibitors (169, 170).

Transforming immunosuppressive apoptotic cells into immunogenic necroptotic cells also presents a promising strategy. Recent studies show that the ERK5 inhibitor XMD8-92 significantly impairs macrophage clearance of apoptotic tumor cells, leading to a higher conversion of apoptotic cells to necroptosis and the release of TAAs. This shift transforms a “cold” tumor microenvironment into a “hot” one, enhancing the immune system’s ability to recognize and attack the tumor. Additionally, ERK5 inhibition drives macrophages to switch from an M2 to an M1 phenotype, accompanied by reduced IL-10 and increased TNF-α secretion, further amplifying anti-tumor immune responses (136).

While RIPK1 and RIPK3 are crucial upstream regulators of necroptosis, clinical studies suggest they may not serve as ideal therapeutic targets. In contrast, targeting the terminal executor of necroptosis, MLKL, may yield more effective therapeutic outcomes by reducing interference with other RCD pathways and minimizing side effects on normal cells (171–174). However, Liao et al. discovered that although necroptosis suppresses tumor growth through MLKL overexpression, in some cases, necroptosis activation can promote tumor metastasis, particularly during the early stages of liver metastasis in PDAC. This paradox arises because necroptosis upregulates the “don’t eat me” signal CD47 on tumor cells, enabling them to evade immune surveillance. A potential strategy to counter this involves combining CD47 blockade with MLKL inhibitors (GW806742X) (75). Interestingly, while MLKL expression is typically associated with poor prognosis, studies on cholangiocarcinoma reveal a positive correlation between MLKL-activated necroptosis and favorable immune cell features, including elevated PD-L1 expression. Patients with high MLKL levels often experience better survival outcomes (79). This finding underscores new opportunities for combining necroptosis-based therapies with immune checkpoint inhibitors. Consequently, developing personalized treatment strategies that tailor necroptosis activation to the tumor type and stage—while integrating immunomodulators, radiotherapy, and other therapies—may help maximize necroptosis’s anti-tumor potential while minimizing adverse effects. This approach holds promise for further investigation in clinical trials.

### 4.2 Targeting TAMs: integration with RCD

M1-type macrophages demonstrate considerable anti-tumor immune activity and directly induce apoptosis and ferroptosis in tumor cells through the secretion of ROS and NO. Furthermore, these cells have the ability to release pro-inflammatory cytokines, including IL-1β and IL-18, which are key activators of pyroptosis (85, 175, 176). In contrast, M2 macrophages have been shown to inhibit ferroptosis through various pathways, ultimately supporting tumor cell survival. Therefore, modulating macrophage activity presents a promising strategy to induce RCD in tumors and reshape the tumor microenvironment to enhance therapeutic efficacy.

#### 4.2.1 Reprogramming TAMs from M2 to M1 phenotype

**Figure 6:**
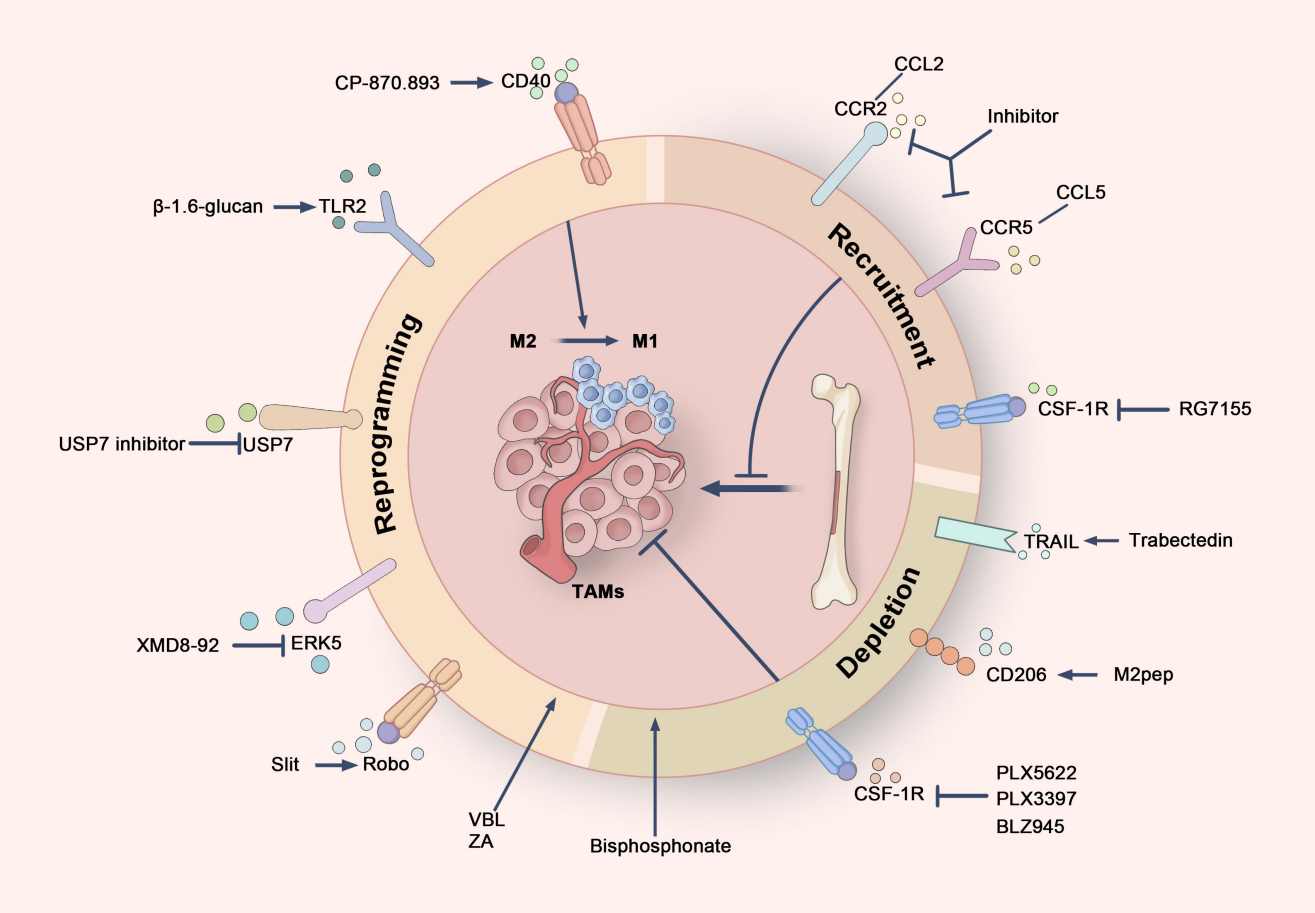
Cancer treatment strategies targeting TAMs. Therapeutic strategies targeting TAMs can be broadly divided into three primary approaches. Firstly, TAMs can be reprogrammed from an immunosuppressive M2 phenotype to a pro-inflammatory M1 phenotype by targeting specific receptors, such as CD40, TLR2, Robo, ERK5, and USP7, thereby boosting anti-tumor immune responses. Secondly, the recruitment of TAMs can be inhibited by blocking critical signaling pathways, including the CSF-1/CSF-1R axis and the chemokine axes CCL2/CCR2 and CCL5/CCR5, which limits the migration of monocytes and macrophages to the tumor microenvironment. Finally, the selective targeting of TRAIL receptors, CD206 receptors, and CSF-1R allows for the depletion of TAMs, particularly M2-type TAMs, thus disrupting their tumor-supportive functions.

Due to their plasticity, M2 macrophages can be reprogrammed into M1 macrophages in response to specific stimuli, providing a potential strategy for tumor therapy (**Figure 6**). Several drugs have been identified that facilitate this reprogramming. Zoledronic acid (ZA), a third-generation nitrogen-containing bisphosphonate (N-BP), is widely utilized in cancer treatment (177). ZA transforms TAMs from M2 to M1 by reducing the production of IL-10, VEGF, and MMP-9, while simultaneously activating NF-κB to restore iNOS and NO expression (178, 179). Recent studies further indicate that when used in combination with PD-1 antibodies, ZA enhances M1 polarization through ferroptosis pathways, thereby improving anti-tumor immunity and the efficacy of immunotherapy​​ (180). Additionally, the anti-tumor agent vinblastine (VBL) induces M1 polarization in TAMs through NF-κB activation and Cyba-dependent ROS generation, significantly increasing macrophage phagocytic activity (181). The CD40 agonist monoclonal antibody CP-870,893 has also shown promising results across various tumor clinical trials (182). By binding to CD40 on macrophages, CP-870,893 activates NF-κB and MAPK pathways, enhancing antigen presentation, promoting pro-inflammatory responses, and driving M1 polarization to strengthen anti-tumor immunity (183–185). Furthermore, CP-870,893 can directly induce apoptosis in CD40-expressing tumor cells​ (184), and has been successfully combined with gemcitabine and cobimetinib in pancreatic cancer immunotherapy, helping to overcome resistance to conventional treatments​​ (186, 187).

In addition to clinically applied drugs, several emerging agents show the ability to reprogram TAMs to the M1 phenotype. Cheng et al. demonstrated that β-1,6-glucan, derived from *Cordyceps militaris*, activates the NF-κB and MAPK pathways via TLR2, effectively converting M2 macrophages into M1 macrophages. This reprogramming also promotes tumor cell apoptosis by cleaving apoptotic proteins such as PARP and caspase-3 (188). Similarly, natural extracts, including polysaccharides from maca and their cationic derivatives (C-MPW), have been found to induce TAM M1 polarization by activating NF-κB, STAT1, and STAT3 signaling pathways. When combined with the chemotherapeutic agent doxorubicin, maca polysaccharides not only enhance anti-tumor efficacy but also significantly inhibit tumor metastasis (176). Slit2, a member of the Slit protein family, exhibits tumor-suppressive activity in various cancers. Research indicates that Slit2 binds to Robo receptors, inhibiting the NF-κB signaling pathway and reducing IL-6 expression (189). This decrease in IL-6 promotes M1 macrophage polarization, restores their phagocytic capacity, and alleviates the inhibitory effects of IL-6 on tumor cell apoptosis, thereby enhancing anti-tumor activity (60, 189). USP7, an important deubiquitinating enzyme involved in regulating DNA degradation and epigenetic modifications, is widely expressed in M2 TAMs and represents a potential therapeutic target. Inhibition of USP7 activates the p38 MAPK pathway, reprogramming M2 macrophages to M1, increasing CD8+ T cell infiltration, and reducing PD-L1 expression in tumor cells, thereby strengthening anti-tumor immune responses (190). In another study, the Extracellular Signal-Regulated Kinase 5 (ERK5) inhibitor XMD8-92 significantly promotes M1 macrophage polarization by inhibiting IL-10 secretion and increasing TNF-α levels. Additionally, ERK5 inhibition reduced the macrophage-mediated clearance of apoptotic tumor cells, leading to the release of tumor antigens and converting a cold tumor microenvironment into a hot one, thus enhancing immune recognition and attack on tumors (136).

#### 4.2.2 TAM depletion strategies

The *in vivo* depletion of TAMs has been shown to effectively reduce CD8+ T cell exhaustion in tumors, thereby restoring their effector functions—a strategy that has been widely studied and validated (191) (**Figure 6**). Trabectedin, an approved anti-cancer drug for soft tissue sarcoma and ovarian cancer, not only inhibits cancer cell proliferation through transcriptional regulation but also selectively induces apoptosis in TAMs via TRAIL receptors within the tumor microenvironment. This reduces the number of TAMs and inhibits metastasis-promoting factors such as CCL2 and CXCL8. Trabectedin’s unique mechanism offers valuable insights into the combination of TAM depletion with direct anti-tumor effects​ (192, 193).

Another promising TAM depletion strategy involves the pro-apoptotic peptide M2pep, which selectively targets the CD206 receptor expressed on M2-type TAMs, sparing other leukocytes. Experimental results demonstrate that M2pep significantly reduces the number of M2 TAMs and prolong survival in tumor-bearing mice, even when administered without additional anti-cancer drugs (194)​. M2pep is currently being explored extensively in nanocarrier systems, particularly in tumor immunotherapy, to modulate the tumor microenvironment and enhance the targeting and therapeutic efficacy of drug delivery​ (195, 196).

Bisphosphonates have also emerged as an effective approach to TAM depletion. Once engulfed by macrophages, these drugs inhibit the function of farnesyl diphosphate (FPP) synthase and induce macrophage apoptosis by suppressing the isoprenylation of RAS-related proteins (197). In breast cancer mouse models, long-term use of zoledronic acid not only reduces TAM numbers but also inhibits angiogenesis and extends survival. Multiple clinical trials have demonstrated the efficacy of bisphosphonates, particularly in postmenopausal breast cancer patients (198).

CSF-1R inhibitors represent another important strategy for eliminating TAMs. By blocking the binding of CSF-1 to its receptor, these inhibitors suppress TAM proliferation and differentiation (199, 200). The utilization of CSF-1R inhibitors, such as PLX5622 and PLX3397, has been demonstrated to diminish the numbers of TAMs and FOXP3+ regulatory T cells within the TME while increasing CD8+ T cell infiltration at both primary and metastatic tumor sites, leading to improved survival in mouse models of osteosarcoma and medulloblastoma​​ (201, 202). Preclinical models suggest that combining CSF-1R inhibitors with other therapies offers significant synergistic effects. For example, in mouse models of lung squamous cell carcinoma and colon cancer, combining the CSF-1R kinase inhibitor PLX3397 with PD-1 inhibitors not only reduced M2 macrophage numbers but also increased CD8+ T cell infiltration, significantly enhancing the anti-tumor efficacy of PD-1/PD-L1 immunotherapy​ (203, 204). In medulloblastoma studies, Fang et al. demonstrated that combining the CSF-1R inhibitor BLZ945 with magnetic hyperthermia could more effectively reduce the number of M2 macrophages in the TME and significantly inhibit tumor growth and recurrence (205)​. Furthermore, targeting CSF-1 with monoclonal antibodies (mAbs) or PLX3397 has been shown to augment the efficacy of radiotherapy by reducing macrophage infiltration, delaying tumor growth in breast cancer mouse models (206).

#### 4.2.3 Inhibition of TAM recruitment and differentiation

In various solid tumors, including primary breast cancer, high expression of macrophage-associated markers within tumor tissue often indicates poor clinical prognosis. Consequently, a principal approach for reducing the number of TAMs is to impede their recruitment to tumor sites (**Figure 6**). Current research primarily focuses on inhibiting specific chemokine and receptor axes, such as the CCR5-CCL5 and CCR2-CCL2 pathways, to diminish macrophage migration. Furthermore, the interference with CSF-1 or its receptor CSF-1R has been shown to significantly decrease TAM accumulation (207, 208).

CCL2 is an indispensable chemokine that orchestrates the infiltration of monocytes expressing high levels of CCR2 into the TME during TAM recruitment (209). CCR2 is closely linked to tumor-related inflammation and can accelerate tumor growth. Preclinical studies have demonstrated that CCL2/CCR2 antagonists effectively inhibit TAM recruitment. Furthermore, CCL2 promotes the recruitment of CCR2-positive regulatory T cells (Tregs), thereby enhancing the efficacy of immunotherapy in breast cancer. In mouse models, the use of neutralizing antibodies to block CCL2 not only reduced the infiltration of bone marrow-derived monocytes and macrophages but also significantly decreased lung metastasis (210). Blocking CCL2 has also been shown to slow the progression of TNBC by inhibiting the renewal of tumor stem cells and preventing TAM polarization toward the M2 phenotype (207, 208).

In addition to the CCL2/CCR2 axis, the CCL5/CCR5 axis serves as a crucial mediator of TAM recruitment. Tumor-secreted CCL5 can bind to CCR5 on macrophages, activating downstream STAT3 and AKT signaling pathways, which enhances the attraction of TAMs and promotes their polarization to the M2 phenotype (211, 212). Moreover, CCL5 is involved in the attraction and differentiation of T lymphocytes, significantly impacting the tumor immune microenvironment. In mouse models of estrogen receptor-positive breast cancer (ER+ BC), the use of neutralizing antibodies against CCL5 significantly reduced macrophage infiltration and decreased tumor volume (213).

Another important therapeutic target is the CSF-1/CSF-1R signaling pathway, which is instrumental to the survival and recruitment of TAMs. Inhibiting this pathway can significantly reduce TAM infiltration in tumors, thereby suppressing tumor progression and metastasis. For example, emactuzumab (RG7155) is an innovative humanized antibody that targets CSF-1R and blocks the recruitment of TAMs. Clinical studies have demonstrated that RG7155 significantly reduced the number of CSF-1R-positive TAMs in tumor biopsies during treatment (214, 215). Additionally, this antibody has shown encouraging clinical progress in diffuse giant cell tumors characterized by CSF-1 overexpression and a large accumulation of TAMs (199).

## 5 Conclusions and future perspectives

In this review, we explored the complex interactions between TAMs and RCD within the tumor microenvironment. TAMs play a crucial role not only in tumor progression but also in influencing the responses to various RCD subtypes, thereby altering the tumor immune microenvironment. By modulating the polarization state and number of TAMs, we can enhance anti-tumor immune responses and improve treatment outcomes. Furthermore, leveraging cytokines and DAMPs released from non-apoptotic forms of RCD can induce tumor cell death while enhancing the infiltration of immune cells in the tumor immune microenvironment and their response to immunotherapy. Future research should focus on several key areas: First, it is essential to gain a deeper understanding of the specific mechanisms by which different RCD subtypes influence the polarization and function of TAMs, particularly in the context of various tumor types and treatment regimens. Second, the development of more tumor-specific cell death-inducing agents that minimize side effects on normal tissues should be prioritized, along with strategies that combine TAM targeting with RCD induction for synergistic effects. Additionally, the identification of new biomarkers to better predict TAM states and their responses to treatment will aid in the personalization of cancer therapy. There should be a strong encouragement for clinical trials involving combination therapies to evaluate their efficacy and safety, providing further insights for future in-depth studies and benefiting a broader population of cancer patients.
